# Correction: The Diagnostic Accuracy of Ultrasonography versus Endoscopy for Primary Nasopharyngeal Carcinoma

**DOI:** 10.1371/journal.pone.0099679

**Published:** 2014-06-02

**Authors:** The PLOS ONE Staff

## Notice of Republication

This article was republished on May 16, 2014 to include figures that were removed during the typesetting process. Please download this article again to view the correct version. The originally published, uncorrected article and the republished, corrected article are provided here for reference.

## Supporting Information

File S1 Originally published, uncorrected article.
(PDF)

File S2 Republished corrected article.
(PDF)
