# Supplementary material for: Correction: The Diagnostic Accuracy of Ultrasonography versus Endoscopy for Primary Nasopharyngeal Carcinoma
Source: PLoS One. 2014 Jun 2;9(6):e99679. doi: 10.1371/journal.pone.0099679 (PMC4041826; doi:10.1371/journal.pone.0099679)
Supplement: File S1 — Originally published, uncorrected article. (PDF) [file pone.0099679.s001.pdf]

The Diagnostic Accuracy of Ultrasonography versus Endoscopy for Primary Nasopharyngeal Carcinoma

Yong Gao[1], Jun-Jie Liu[1], Shang-Yong Zhu[1]*, Xiang Yi[2]

1 Department of Ultrasonography, First Affiliated Hospital of Guangxi Medical University, Nanning, Guangxi, China, 2 Department of Otolaryngology, First Affiliated Hospital of Guangxi Medical University, Nanning, Guangxi, China

Abstract

Objective: To compare the accuracy of ultrasonography (US) with the current clinical standard of endoscopy for a diagnosis of nasopharyngeal carcinoma (NPC).

Methods: A total of 150 patients suspected of having NPC underwent US and endoscopy. A diagnosis was obtained from an endoscopic biopsy collected from each suspected tumor and was compared with a biopsy obtained from a normal nasopharynx. The diagnostic accuracy of US and endoscopy for NPC was evaluated using receiver operating curve (ROC) analysis performed by MedCalc Software.

Results: The sensitivity, specificity, and accuracy of US versus endoscopy for this cohort were 90.1%, 84.8%, and 87.3% for US, and 88.7%, 97.5%, and 93.3% for endoscopy, respectively. Both US and endoscopy exhibited good diagnostic accuracy for NPC with area under the curve (AUC) values of 0.929 and 0.938, respectively. However, this difference was not significant ($Z = 0.36$, $P = 0.72$).

Conclusion: US is a useful tool for the detection of tumors in endoscopically suspicious nasopharynx tissues, and also for the detection of subclinical tumors in endoscopically normal nasopharynx tissues.

Citation: Gao Y, Liu J-J, Zhu S-Y, Yi X (2014) The Diagnostic Accuracy of Ultrasonography versus Endoscopy for Primary Nasopharyngeal Carcinoma. PLoS ONE 9(3): e90412. doi:10.1371/journal.pone.0090412

Editor: Pierre Busson, Institute of Cancerology Gustave Roussy, France

Received November 21, 2013; **Accepted** February 1, 2014; **Published** March 3, 2014

Copyright: © 2014 Gao et al. This is an open-access article distributed under the terms of the Creative Commons Attribution License, which permits unrestricted use, distribution, and reproduction in any medium, provided the original author and source are credited.

Funding: This work was supported by Guangxi Science Fund Project, grant number: 0832125. The funders had no role in study design, data collection and analysis, decision to publish, or preparation of the manuscript.

Competing Interests: The authors have declared that no competing interests exist.

* E-mail: zhushangyong2013@sina.com

Introduction

Nasopharyngeal endoscopy is typically used to detect nasopharyngeal carcinoma (NPC). A definitive diagnosis is subsequently confirmed with an endoscopic biopsy of the primary tumor site [1]. In general, there are five NPC phenotypes: nodular, cauliflower-type, submucosal, infiltrating, and ulcerated. Since a biopsy can only sample a small fraction of the nasopharynx, it is possible that small mucosal, submucosal, or infiltrating tumors may go undetected [2]. Therefore, it is recommended that patients with such tumors undergo random endoscopic biopsies to sample the nasopharynx [3]. Correspondingly, the diagnostic potential of less invasive and more patient-friendly imaging modalities have been investigated in recent years [4,5]. For example, both computed tomography (CT) and magnetic resonance imaging (MRI) of the head and neck have been used for the diagnosis and staging of NPC [4,5,6]. However, the latter is preferred based on its ability to delineate small anatomical structures that constitute the boundary of the nasopharynx. In addition, MRI is better able to map the extent of the tumor that is present in the skull base, the paranasal sinuses, and the brain, while also discriminating between the primary tumor and adjacent retropharyngeal nodes [5]. Despite these advantages, however, MRI is not widely accessible, particularly in more remote regions of the world.

A previous study indicated that ultrasonography (US) may be a useful tool for diagnosing NPC and for defining the relationship between a tumor and the parapharyngeal space [7]. By using the parapharyngeal space and parotid gland as an acoustic window, along with the strong echo of the nasopharynx gas as a boundary, US has been found to provide anatomic details of the nasopharynx, including mucosal changes. Specifically, US has been shown to distinguish the normal anatomy of the nasopharynx and parapharyngeal space, the presence of NPC, and the extent of NPC in the parapharyngeal space [7]. The latter is usually suspected if the echo of the soft tissue is interrupted or distorted, if the tumor is distorted or surrounded by the internal carotid artery, if the acoustic shadow of the styloid process disappears, or if the margin of the parotid gland is inseparable from the tumor [7]. However, due to the concern that imaging studies may not detect small mucosal tumors, US imaging has not been validated as an initial diagnostic test for NPC. Thus, the aim of this prospective study was to compare the accuracy of US with endoscopy for suspected cases of NPC, and to evaluate whether US can detect subclinical cancers that are not detected by endoscopy.

Materials and Methods

Patients

This study protocol was approved by the Guangxi Medical University ethics committee and written informed consent was obtained from all patients. Patients suspected of having NPC were recruited to this prospective study between January 2010 and January 2013 in a region where NPC is endemic. Suspicion of NPC was based on the presence of metastatic cervical lymph nodes and/or a nasopharyngeal abnormality accompanied by nonspecific symptoms (e.g., epistaxis, nasal obstruction, hearing loss), and/or positive Epstein-Barr virus (EBV) serologic results. Patients were excluded if they did not successfully undergo US, endoscopy, and an endoscopic biopsy, or if a non-NPC tumor of the nasopharynx was diagnosed. The study group included 150 patients (99 males, 51 females) ranging in age from 21–68 y (mean, 48 y). US examination was performed prior to the nasopharyngeal endoscopy and endoscopic biopsy to ensure that the biopsy would not affect nasopharynx imaging. In addition, an endoscopy was performed following an endoscopic biopsy, and was performed with knowledge of the clinical reasons for suspecting NPC.

US Examinations and Image Analysis

US was performed using an Esaote Technos MPX or MP scanner (Esaote, Genoa, Italy) with a 3.5–5.0 MHz convex-array transducer or a 7.0–13.0 MHz linear-array transducer for obese patients versus thin patients, respectively. Patients were placed in the supine position with the neck biased toward the opposite side and slightly tilted back. The transducer was placed between the mastoid and mandible ramus aspect of the neck, and the nasopharynx and parapharyngeal space were examined in transverse, longitudinal, and oblique planes. In our experience, the parotid gland can be used as an acoustic window. Therefore, the operator subsequently requested that patients swallow to confirm the linear air in the nasopharynx and pharyngeal recess. US images obtained for each patient were acquired, reviewed, and interpreted by two sonologists (Y.G., S.Y.Z) with 8 y and 24 y of US experience, respectively. Each scan was scored from 1 to 4 (Table 1). For the purpose of this study, scores of 1 and 2 were considered negative for NPC and scores of 3 and 4 were considered positive for NPC. All interpretations were performed prior to the collection of endoscopic biopsies, and the readers were blinded to each patient's final diagnosis. Prior to the study, the methods of image acquisition and interpretation were determined and decisions regarding the findings were reached by consensus.

Endoscopy and Endoscopic Biopsy

An endoscopy was performed after each US examination. This procedure was performed with knowledge of the clinical reasons for suspecting NPC, although previous US findings were not provided. The absence of NPC was defined as normal endoscopic findings or findings that showed a minor abnormality not suspicious of NPC. In contrast, the presence of NPC was defined as suspicious abnormalities or definitive NPC. An endoscopic biopsy was performed at the site of abnormalities. Patients with an endoscopically normal nasopharynx underwent endoscopic sampling biopsies from both the right and left sides of the posterior wall of the nasopharynx. Sampling specimens were selected for microscopic examination and underwent processing for hematoxylin-eosin staining.

Statistical Analysis

MedCalc software (version 9.2.0.0; Broekstraat, Mariakerke, Belgium) was used for statistical analyses. Sensitivity, specificity, negative predictive value, positive predictive value, and accuracy of US and endoscopy were also calculated. The diagnostic accuracy of US and endoscopy for NPC was evaluated using receiver operating characteristic (ROC) analysis. Area under the curve (AUC) values less than 0.7, between 0.7 and 0.9, or greater than 0.9 were considered to indicate low, medium, and high diagnostic accuracies, respectively. A Z-value was also calculated using MedCalc software, and P-values less than 0.05 were considered statistically significant.

Results

Pathologic diagnosis

Of the patients analyzed by US and endoscopy, 79/150 (52.7%) were negative for NPC and 71/150 (47.3%) were positive for NPC. All of the NPC cases involved non-keratinizing undifferentiated carcinomas, with 10/71 (14.1%) being submucosal tumors and 16/71 (22.5%) being infiltrating tumors. Among the non-NPC patients, nasal melanoma (n = 1), lymphoma (n = 1), and benign mucosal lesions (n = 5) were identified, while the remaining patients were healthy.

US detection of NPC

US detected NPC in 64/71 (90.1%) patients and NPC was excluded for 67/79 (84.8%) patients (Table 2). US was able to detect tumors that caused an obvious focal mass in the nasopharynx, and early tumors that produced only mild thickening of the mucosa. US was also used to measure tumor volume and to characterize tumor boundaries, tumor shape, internal echo patterns, and the parapharyngeal space. Tumor diameters were found to range from 1 to 5 cm, and 38 tumors had invaded the parapharyngeal space. However, US did not detect NPC in 7/71 (9.8%) patients that did have NPC, and US mistakenly detected

Table 1. US Imaging Criteria used to Grade the Appearance of the Nasopharynx.

Grade	Appearance
Grade 1, normal	Symmetrical mucosal thickening<3 mm
Grade 2, low suspicion of NPC	A smooth band of symmetrically thickened mucosa>3 mm in depth
Grade 3, high suspicion of NPC	Asymmetry between the right and left sides of the nasopharynx. or an obvious focal mass present in the nasopharynx
Grade 4, NPC	Asymmetry between the right and left sides of the nasopharynx, or an obvious focal mass present in the nasopharynx accompanied by invasion of the parapharyngeal space

Note: Grades 1 and 2 indicate patients without NPC; grades 3 and 4 indicate patients with NPC.
doi:10.1371/journal.pone.0090412.t001

Table 2. Accuracy of US versus Endoscopy for this Cohort (n = 150*).

Parameter	US	Endoscopy
True-positive finding (n)	64	63
True-negative finding (n)	67	77
False-positive finding (n)	12	2
False-negative finding (n)	7	8
Sensitivity (%)	90.1	88.7
Specificity (%)	84.8	97.5
Positive predictive value (%)	84.2	96.9
Negative predictive value (%)	90.5	90.6
Accuracy (%)	87.3	93.3

*: 71 patients were positive for NPC and 79 patients were negative for NPC.
doi:10.1371/journal.pone.0090412.t002

cancer in 12/79 (15.2%) patients with asymmetrical mucosal abnormalities caused by benign lymphoid hyperplasia.

Endoscopic detection of NPC

Endoscopy detected NPC in 63/71 (88.7%) patients and NPC was excluded for 77/79 (97.5%) patients (Table 2). Furthermore, endoscopy did not detect NPC in 8/71 (11.3%) patients that did have NPC. Two of these patients had small cancers involving the pharyngeal recess, and six patients had cancers beneath the nasopharyngeal mucosa. NPC was mistakenly diagnosed by endoscopy in 2/79 (2.5%) patients with asymmetrical mucosal abnormalities caused by benign lymphoid hyperplasia.

Comparison of US and Endoscopy

US detected all ten cases of submucosal cancers, resulting in a detection rate of 100.0%. In contrast, endoscopy detected 4/10 of the submucosal cases, resulting in a detection rate of 40.0%. For the 16 cases involving infiltrative tumors, US detected 15/16 (93.8%) of these cases, while endoscopy detected 14/16 (87.5%) of these cases. There were eight patients with NPC that were not detected by endoscopy, while six of these cancers were identified using US (four cases involved nasopharyngeal submucosal masses and two cases involved infiltration of the nasopharyngeal mucosa). Conversely, there were seven patients with NPC that were not detected by US, while five of these cancers were identified using endoscopy. These five cases included two anterior nasopharynx masses, two slightly plump nasopharynx pharyngeal recesses, and in one case, the top surface of the nasopharyngeal mucosa was rough. The final two false-negative findings were confirmed using random endoscopic biopsies that sampled the nasopharynx. The sensitivity, specificity, negative and positive predictive values, and accuracy values associated with the use of US and endoscopy are listed in Table 2. Both US and endoscopy achieved a good diagnostic accuracy for NPC with AUC values of 0.929 and 0.938, respectively (Figure 1). Moreover, there was no statistically significant difference in the diagnostic accuracy for NPC between US and endoscopy ($Z = 0.36$, $P = 0.72$). Overall, US was able to detect tumors in endoscopically suspicious nasopharynx tissues, and was also able to detect subclinical tumors in endoscopically normal nasopharynx tissues. Representative patient images of concordant and discordant results are shown(Figures 2–5).

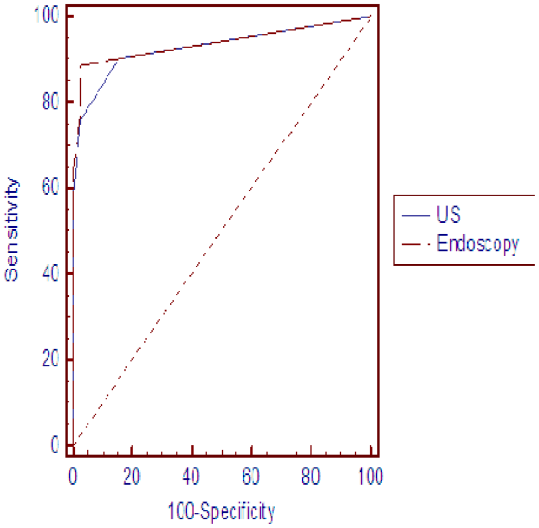

Figure 1. Diagnostic accuracy of US and endoscopy using ROC analysis for this cohort (n = 150).
doi:10.1371/journal.pone.0090412.g001

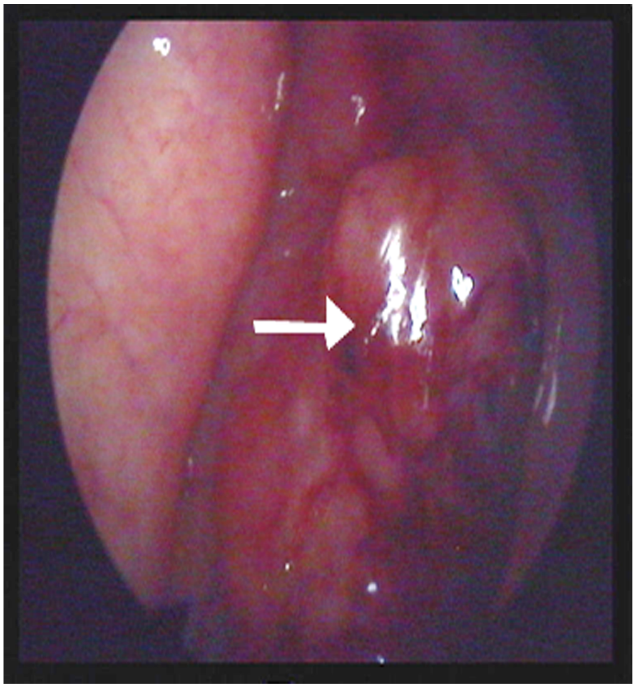

Figure 2. A 60-year-old male with NPC underwent US and endoscopy. A large focal mass was detected on the right side of the nasopharynx by both US and endoscopy. A: An US image obtained by applying a 3.5–5.0 MHz convex-array transducer to the oblique plane. The large focal mass present in the nasopharynx is indicated with an arrow (grade 3). B: An US image of the transverse plane also showed a mass present (indicated with arrow). C: An US image scanned from the left detected normal nasopharynx tissue. The parotid gland (PG) provided an acoustic window with air from the upper pharyngeal recess (indicated with arrowhead) descending to the nasopharynx (indicated with curved arrow). D: An endoscopy image of a focal mass (indicated with arrow) present in the nasopharynx.
doi:10.1371/journal.pone.0090412.g002

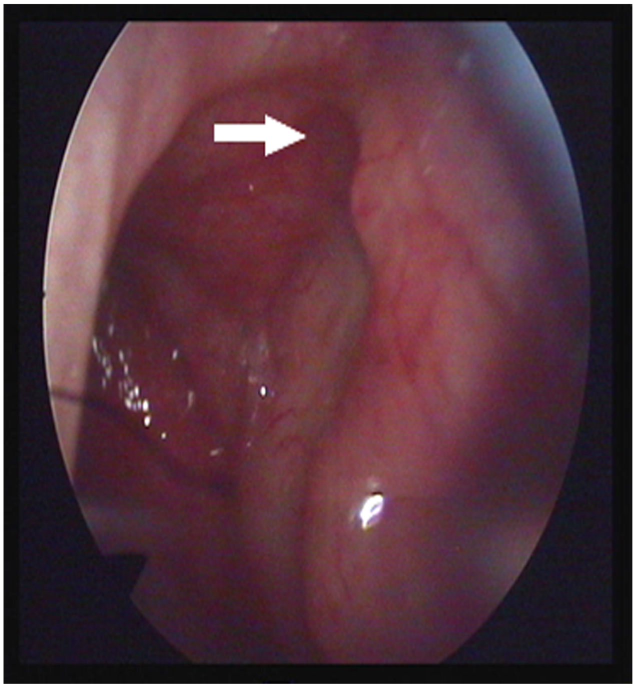

Figure 3. A 35-year-old female with NPC that was confined to the mucosa of the left pharyngeal recess underwent US and endoscopy. A: An oblique US image obtained using a 3.5–5.0 MHz convex-array transducer showed that the tumor caused a focal mass (indicated with arrow) in the pharyngeal recess (US grade 3). B: An endoscopy image also showed a focal mass (indicated with arrow) present in the pharyngeal recess.
doi:10.1371/journal.pone.0090412.g003

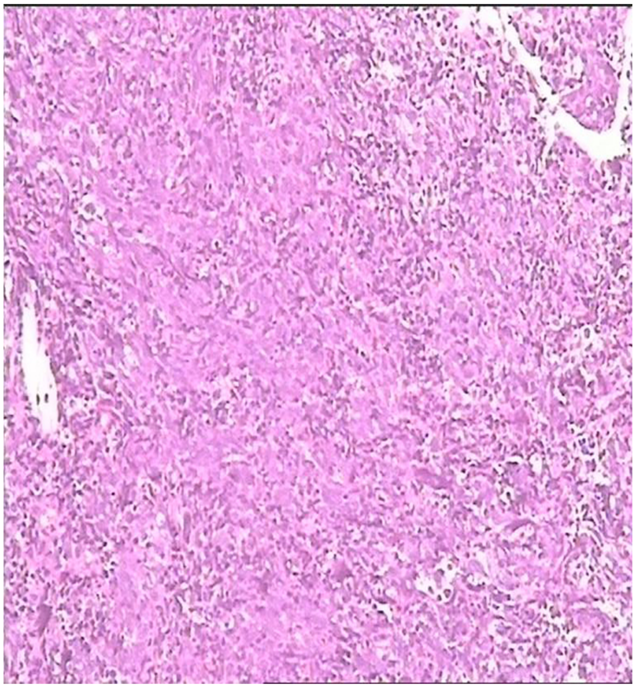

Figure 4. A 45-year-old female with NPC underwent US and endoscopy. A: An oblique US image obtained using a 3.5–5.0 MHz convex-array transducer is shown. The tumor had infiltrated (indicated with arrow) the nasopharynx (US grade 3). B: An endoscopy image showed the pharyngeal recess to be slightly rough (indicated with arrow). C: A non-keratinizing undifferentiated carcinoma confirmed by pathology.
doi:10.1371/journal.pone.0090412.g004

Discussion

For this cohort, US was able to detect primary NPCs that caused an obvious focal mass, deeply infiltrating tumors, and early tumors that produced mild thickening of the mucosa. Furthermore, US achieved a good diagnostic accuracy for NPC with an AUC value of 0.929. A similar diagnostic sensitivity and specificity were identified for both US and endoscopy methods (90.1% and 84.8% for US, and 88.7% and 97.5% for endoscopy, respectively in each case), and therefore, a significant difference in diagnostic accuracy for the two modalities was not observed ($Z = 0.36$, $P = 0.72$).

It was previously reported that 10% of cancers are missed at endoscopy, with the majority of these missed tumors being small or deeply infiltrating tumors that often involve the submucosa [3]. In the present study, the tumor detection rate for US was higher than that for endoscopy when submucosal or infiltrative tumors of NPC were present (100% vs. 40%, and 93.8% vs. 87.5%, respectively in each case). These tumor types exhibit endogenous growth that may directly invade the submucosa or intracalvarium, resulting in a smooth and symmetrical mucosal phenotype. Typically, mucosal structure can be clearly observed using US. Of the eight patients with NPC that were not detected by endoscopy in the present study, four cases involved nasopharyngeal submucosal masses and two cases involved infiltration of the nasopharyngeal mucosa, and these cases were detected by US. Small tumors can often be identified with US based on mucosal thickening, with symmetrical thickening typically associated with benign diseases and asymmetric thickening associated with cancers in their early stages.

Given that an endoscopic biopsy is an invasive procedure, patients with an endoscopically normal nasopharynx, or their clinicians, may be reluctant to undergo or repeat this procedure due to discomfort, risk of bleeding, and the potential administration of a general anesthetic. Consistent with the results of previous studies [8,9], sampling biopsies obtained from endoscopically normal nasopharynx tissues were found to improve NPC detection rates. However, even under these circumstances, tumors were still missed using the current clinical reference standards. Although, the results of the present study do indicate that US could be used to guide biopsies for the detection of tumors in endoscopically normal nasopharynx tissues.

In the present study, US was found to facilitate the detection of subclinical tumors in endoscopically normal nasopharynx tissues, as well as tumors present in endoscopically suspicious nasopharynx tissues. The adenoids that are located in the central roof and upper posterior wall of the nasopharynx are a common site for benign diseases. Moreover, it has previously been shown in other endoscopy-based studies [3] that adenoidal lymphoid hyperplasia can be mistaken for NPC. However, in the present study, US was able to distinguish benign adenoids and malignant masses. US could also be used to examine invasion of the parapharyngeal space to distinguish between benign and malignant tumors based on the following findings: if an echo of the soft tissue is interrupted or distorted, if a tumor is distorted or surrounded by the internal carotid artery, if an acoustic shadow of the styloid process disappears, or if the margin of the parotid gland is inseparable from the tumor. These observations could indicate NPC invasion of the parapharyngeal space. However, even without this knowledge, a simple comparison of mucosal thickness on the

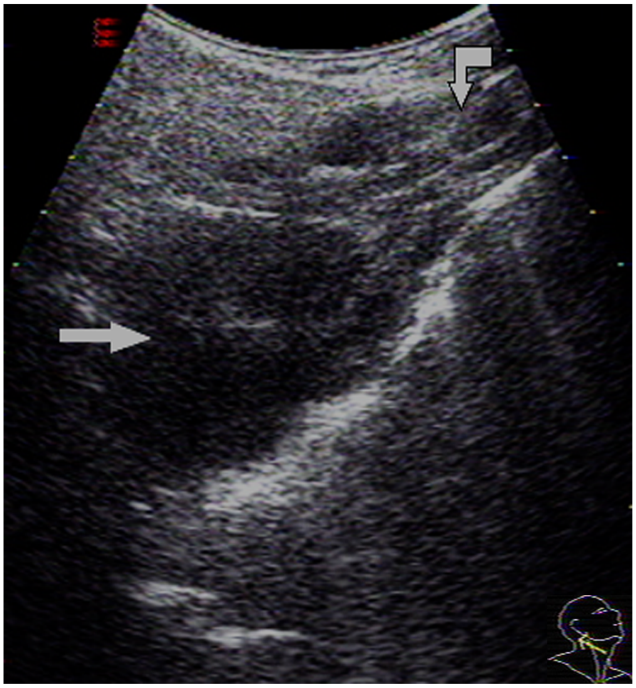

Figure 5. An US image obtained using a 3.5–5.0 MHz convex-array transducer applied to the oblique plane of a 75-year-old female with NPC that was not detected by endoscopy. A NPC (grade 4) with a submucosal component (indicated with arrow) that had deeply invaded the parapharyngeal space (indicated with curved arrow) was detected.
doi:10.1371/journal.pone.0090412.g005

right versus the left side of the nasopharynx could be used to successfully distinguish generalized symmetrical thickening associated with benign disease from asymmetric thickening of an early tumor. Correspondingly, the use of US to indicate potentially malignant lesions could be used to identify patients who should undergo a repeat biopsy. Furthermore, it is possible that US could identify the best site for a repeat biopsy, while also gauging the appropriate and safest depth for the biopsy. These are important considerations for cancers that are in close proximity to the internal carotid artery, and thus are associated with a higher risk for biopsy procedures.

It is important to note that the results of the present study indicate that US should not replace an endoscopy. For example, of the seven patients with NPC that were detected by endoscopy and not by US, these cases involved very small nasopharyngeal tumors present on the top wall which only exhibited a rough nasopharyngeal mucosal surface by endoscopy. Therefore, very small lesions that do not exhibit a smooth mucosal surface or significant thickening, may be associated with a poor NPC detection rate by US. In contrast, endoscopy can detect early, subtle changes in the mucosal surface. Therefore, it may be more appropriate for US to be applied as an adjunct method to endoscopy for the detection of subclinical cancers present in endoscopically normal nasopharynx tissue. A great effort was also made to assess patients in whom a cancer had been missed during an initial endoscopy, yet was subsequently identified using US. As such, there was a potential for bias toward US in this study. Correspondingly, the true incidence of NPC in this study population may be underestimated, and the sensitivity overestimated. However, it was not the aim of the current study to definitively determine the accuracy of these two techniques. Rather, the aim was to determine the potential benefit of performing US for the examination of endoscopically normal nasopharynx tissue. Lastly, color Doppler was not sufficiently sensitive to detect tumor blood supply, perhaps due to the anatomical location of the nasopharynx deep within the head. Therefore, further study is needed to evaluate the capacity for US to detect a tumor's blood supply and to distinguish benign and malignant tumors.

US can also readily measure tumor volume, can characterize the boundaries, shape, and internal echo of a mass, and can evaluate the relationship between a tumor and the parapharyngeal space. In previous studies, primary tumor volume and invasion of the parapharyngeal space were found to be closely related to NPC survival rates [10,11,12]. Thus, tumor volume and parapharyngeal space invasion may represent prognostic factors that could be incorporated into the existing TNM system based on the accessibility of US for tumor detection [13,14]. However, additional studies will be needed to further evaluate this possibility.

Summary

In conclusion, US achieved a good diagnostic accuracy for NPC and is a less invasive and more patient-friendly technique compared to endoscopy. As such, US could be used for the initial investigation of primary tumors in patients suspected of having NPC, especially when a repeat biopsy is needed for endoscopically normal nasopharynx tissue. Furthermore, for patients with abnormal US results, US could subsequently be used to guide the biopsy of a subclinical tumor site.

Author Contributions

Conceived and designed the experiments: SYZ. Performed the experiments: YG. Analyzed the data: JJL. Contributed reagents/materials/analysis tools: XY. Wrote the paper: YG NO.

References

1. Chan AT, Felip E, ESMO Guidelines Working Group (2009) Nasopharyngeal cancer: ESMO clinical recommendations for diagnosis, treatment and follow-up. Ann Oncol 20: 123–125.
2. King AD, Vlantis AC, Bhatia KS, Zee BC, Woo JK, et al. (2011) Primary nasopharyngeal carcinoma: diagnostic accuracy of MR imaging versus that of endoscopy and endoscopic biopsy. Radiology 258: 531–537.
3. Wei WI, Sham JS, Zong YS, Choy D, Ng MH (1991) The efficacy of fiberoptic endoscopic examination and biopsy in the detection of early nasopharyngeal carcinoma. Cancer 67: 3127–3130.
4. King AD, Vlantis AC, Tsang RK, Gary TM, Au AK, et al. (2006) Magnetic resonance imaging for the detection of nasopharyngeal carcinoma. AJNR Am J Neuroradiol 27: 1288–1291.
5. Abdel Khalek Abdel Razek A, King A (2012) MRI and CT of nasopharyngeal carcinoma. AJR Am J Roentgenol 198:11–18.
6. King AD, Bhatia KS (2010) Magnetic resonance imaging staging of nasopharyngeal carcinoma in the head and neck. World J Radiol 2:159–165.
7. Pan D, Zhu SY, Xu YB, Wu YF, Lun HM, et al. (2013) Sonographic findings of Nasopharyngeal Carcinoma and its involvement in the parapharyngeal space. J Ultrasound Med 32: 1041–1047.
8. Ng WT, Choi CW, Lee MC, Law LY, Yau TK, et al. (2010) Outcomes of nasopharyngeal carcinoma screening for high risk family members in Hong Kong. Fam Cancer 9: 221–228.
9. Sham JS, Wei WI, Zong YS, Choy D, Guo YQ, et al. (1990) Detection of subclinical nasopharyngeal carcinoma by fibreoptic endoscopy and multiple biopsy. Lancet 335: 371–374.
10. Sarisahin M, Cila A, Ozyar E, Yıldız F, Turen S (2011) Prognostic significance of tumor volume in nasopharyngeal carcinoma. Auris Nasus Larynx 38: 250–254.
11. Lee CC, Chu ST, Ho HC, Lee CC, Hung SK (2008) Primary tumor volume calculation as a predictive factor of prognosis in nasopharyngeal carcinoma. Acta Otolaryngol 128: 93–97.

12. Tang LL, Li WF, Chen L, Sun Y, Chen Y, et al. (2010) Prognostic value and staging categories of anatomic masticator space involvement in nasopharyngeal carcinoma: A study of 924 cases with MR Imaging. Radiology 257: 151–157.
13. Huang PY, Sun ZY, Xie CM, Chen QY, Wen YF, et al. (2012) Prognostic significance of the various classifications for parapharyngeal space involvement in nasopharyngeal carcinoma. Acta Otolaryngol 132: 1197–1207.
14. Zhou JY, Chong VF, Khoo JB, Chan KL, Huang J (2007) The relationship between nasopharyngeal carcinoma tumor volume and TNM T-classification: A quantitative analysis. Eur Arch Otorhinolaryngol 264:169–174.
